# Safety and Immunogenicity of the Recombinant BCG Vaccine AERAS-422 in Healthy BCG-naïve Adults: A Randomized, Active-controlled, First-in-human Phase 1 Trial

**DOI:** 10.1016/j.ebiom.2016.04.010

**Published:** 2016-04-19

**Authors:** Daniel F. Hoft, Azra Blazevic, Asmir Selimovic, Aldin Turan, Jan Tennant, Getahun Abate, John Fulkerson, Daniel E. Zak, Robert Walker, Bruce McClain, Jerry Sadoff, Judy Scott, Barbara Shepherd, Jasur Ishmukhamedov, David A. Hokey, Veerabadran Dheenadhayalan, Smitha Shankar, Lynn Amon, Garnet Navarro, Rebecca Podyminogin, Alan Aderem, Lew Barker, Michael Brennan, Robert S. Wallis, Anne A. Gershon, Michael D. Gershon, Sharon Steinberg

**Affiliations:** a Department of Internal Medicine, Saint Louis University, St. Louis, MO, United States; b Aeras, United States; c Seattle BioMed, United States; d Aurum Institute, South Africa; e Division of Pediatric Infectious Diseases, Columbia University, United States

**Keywords:** Tuberculosis;, Recombinant BCG;, Herpes zoster;, Transcriptomes;, Functional T cell assays

## Abstract

**Background:**

We report a first-in-human trial evaluating safety and immunogenicity of a recombinant BCG, AERAS-422, over-expressing TB antigens Ag85A, Ag85B, and Rv3407 and expressing mutant perfringolysin.

**Methods:**

This was a randomized, double-blind, dose-escalation trial in HIV-negative, healthy adult, BCG-naïve volunteers, negative for prior exposure to *Mtb*, at one US clinical site. Volunteers were randomized 2:1 at each dose level to receive a single intradermal dose of AERAS-422 (> 10^5^–< 10^6^ CFU = low dose, ≥ 10^6^– < 10^7^ CFU = high dose) or non-recombinant Tice BCG (1–8 × 10^5^ CFU). Randomization used an independently prepared randomly generated sequence of treatment assignments. The primary and secondary outcomes were safety and immunogenicity, respectively, assessed in all participants through 182 days post-vaccination. ClinicalTrials.gov registration number: NCT01340820.

**Findings:**

Between Nov 2010 and Aug 2011, 24 volunteers were enrolled (AERAS-422 high dose, *n* = 8; AERAS-422 low dose, *n* = 8; Tice BCG, *n* = 8); all were included in the safety and immunogenicity analyses. All 24 subjects had at least one adverse event, primarily expected local reactions. High dose AERAS-422 vaccination induced Ag85A- and Ag85B-specific lymphoproliferative responses and marked *anti*-mycobacterial activity in a whole blood bactericidal activity culture assay (WBA), but was associated with varicella zoster virus (VZV) reactivation in two vaccinees. These volunteers displayed high BCG-specific IFN-γ responses pre- and post-vaccination possibly predisposing them to autocrine/paracrine negative regulation of immune control of latent VZV. A systems biology transcriptomal approach identified positive correlations between post-vaccination T cell expression modules and WBA, and negative correlations between post-vaccination monocyte expression modules and WBA. The expression of one key macrophage marker (F4/80) was constitutively elevated in the two volunteers with zoster.

**Interpretation:**

The unexpected development of VZV in two of eight healthy adult vaccine recipients resulted in discontinuation of AERAS-422 vaccine development. Immunological and transcriptomal data identified correlations with the development of TB immunity and VZV that require further investigation.

**Funding:**

Aeras, FDA, Bill and Melinda Gates Foundation.

## Highlights

• Novel rBCG first-in-human trial overexpressing 3Mtb Ags induces a more potent human immune response than standard BCG.
• Transcriptomal studies demonstrated correlations between mycobacterial growth inhibition growth and potent T cell responses.
• rBCG was associated with varicella-zoster reactivation and associated with high interferon gamma and EMR1 expression.
  A first-in-human trial evaluating safety and immune responses of a recombinant BCG vaccine was reported. The new vaccine expresses additional TB antigens and escapes from seclusion to induce a better immune response in CD8 T cells. The immune response produced in humans could inhibit the growth of the TB pathogen. Unfortunately the vaccine was associated with shingles and studies were done to explore the reasons why that occurred.

## 1 Introduction

The World Health Organization (WHO) has declared tuberculosis (TB) a global health emergency and a new vaccine is desperately needed. The development of recombinant bacillus Calmette-Guerin (BCG), which improves upon the wild type attenuated *Mycobacterium bovis* strains widely used to prevent the most serious TB disease, is one proposed approach (Horwitz et al., 2000, Velmurugan et al., 2013).

AERAS-422 is a recombinant BCG (rBCG) derived from the Danish 1331 strain of BCG (Sun et al., 2009). AERAS-422 includes a chromosomal gene coding for a mutant perfringolysin (PFO_G137Q_) derived from *Clostridium perfringens*. Phagocytosis of AERAS-422 by human macrophages does not result in cell lysis. PFO_G137Q_ retains the ability to perforate endosomal membranes, allowing antigens to enter the cytosol and thus stimulate an MHC Class I-restricted immune response. AERAS-422 contains a plasmid encoding selected immunodominant antigens expressed by *Mtb* during both active infection (Rv3804c and Rv1886c, aka Ag85A and Ag85B, respectively), and reactivation of latent infection (Rv3407).

AERAS-422 is a genetically stable rBCG capable of priming strong and more persistent CD8 + T cell responses in mice than nonrecombinant BCG or other rBCGs (Sun et al., 2009). Preliminary data from immunodeficient (SCID) mice indicate that AERAS-422 is significantly more attenuated than nonrecombinant BCG (Sun et al., 2009). We report the first administration of AERAS-422 in humans to evaluate safety and immunogenicity.

## 2 Methods

### 2.1 Study Design

A Phase I, randomized, double-blind, dose-escalation study was conducted at Saint Louis University (SLU), following Declaration of Helsinki principles and approved by SLU and Aeras Institutional Review Boards.

### 2.2 Participants

After giving written informed consent, 24 HIV-negative, BCG-naïve, mycobacteria-naïve, 18–40 year old healthy subjects were sequentially assigned to study groups.

### 2.3 Randomization and Masking

The first 12 subjects were randomized 2:1 to receive either AERAS-422 low-dose (≥ 10^5^ to < 10^6^ CFU) or Tice® BCG. After review of day 28 blinded safety data by the principal investigator and local medical monitor, 12 more subjects were randomized to receive either AERAS-422 high-dose (≥ 10^6^ to < 10^7^ CFU) or Tice BCG. All vaccinations were given intradermally. Randomization was according to a randomly generated sequence of treatment assignments, prepared by an independent statistician and provided in a sealed envelope. Doses of vaccine were prepared in identical syringes by the study vaccine manager at the site, who was unmasked to treatment allocation. The volunteers, study staff administering vaccinations or undertaking follow-up clinical assessments, and laboratory staff were masked to treatment assignments.

### 2.4 Procedures

Subjects were followed at 3, 7, 14, 28, 56, 84, 112, 140, and 182 days post-vaccination.

### 2.5 Intracellular Cytokine Staining Assay (ICS) (7-color)

Refer to the Supplementary materials for details.

### 2.6 Whole Blood Lymphoproliferative Assay

Whole blood samples were diluted ten fold with RPMI and expanded with optimal doses of Ag85B peptide pool (15-mers overlapping by 11 amino acids covering the entire protein sequence), rAg85b protein, live BCG, or rested in medium for 7 days at 37 °C in 5% CO_2_. During the last 10–14 h, cultures were pulsed with tritiated thymidine and cell-incorporated radioactivity measured (results are presented as dpm).

### 2.7 Whole Blood Bactericidal Activity (WBA) Against Tice BCG

Mycobactericidal activity was studied in whole blood cultures as previously described (Hoft et al., 2002, Worku and Hoft, 2003, Cheon et al., 2002, Fletcher et al., 2013). Changes in the *log*_10_ viable bacilli from days 0 to 4 were calculated as: *log*_10_ (final/initial). Results were reported as log change per day of whole blood culture. Data management, standard curves, interpolation, and calculation of *log*_10_ CFU were performed using customized computer software from author RSW (full protocol and software available on request).

### 2.8 RNA Collection, Processing, Sequencing, and Statistical Analyses

Refer to the Supplementary materials for details.

### 2.9 Transcription Module Over-representation Analyses

Blood transcriptome module over-representation analyses were performed using module annotations previously provided (Obermoser et al., 2013, Li et al., 2014). Statistical significance of module over-representation was assessed by Fisher's Exact Test, with multiple testing adjustment performed using the Benjamini-Hochberg algorithm. Module-level expression values were computed by averaging over the log2 expression levels of all genes comprising a given module.

### 2.10 Outcomes

The primary and secondary outcomes were safety and immunogenicity, respectively, assessed in all participants through 182 days post-vaccination. The safety profile was assessed by evaluation of solicited and unsolicited AEs through day 56, and serious AEs through day 182. Injection site reactions, axillary tenderness, headache, myalgia, arthralgia, fatigue and temperature were recorded by the subject on diary cards for 7 days after vaccination. Additional safety evaluations are described in the Supplementary materials.

### 2.11 Statistical Analysis

The sample size was selected as adequate for initial review of the safety profile of AERAS-422; the study was not powered to detect differences in AEs between dose groups. All volunteers were included in the safety and efficacy analyses. Friedman repeated-measures analysis of variance (ANOVA) was used to study overall increases after vaccination, the Wilcoxon matched-pairs test was used to identify significantly increased post-vaccination compared with matched pre-vaccination responses, and the Mann-Whitney *U* test was used to compare responses between groups. McNemar's test and Fisher's exact test were used to compare paired and unpaired categorical data, respectively. Analyses were completed with Statistica (Statsoft).

The trial is registered with ClinicalTrials.gov (number: NCT01340820).

### 2.12 Role of the Funding Source

Aeras was the trial sponsor and contributed to study design and data analysis. The other funders had no role in study design, data collection, data analysis, data interpretation, or writing of the report. The corresponding author had full access to all the data in the study and had final responsibility for the decision to submit for publication.

## 3 Results

### 3.1 Study Subjects and Safety Evaluation

**Fig. 1:**
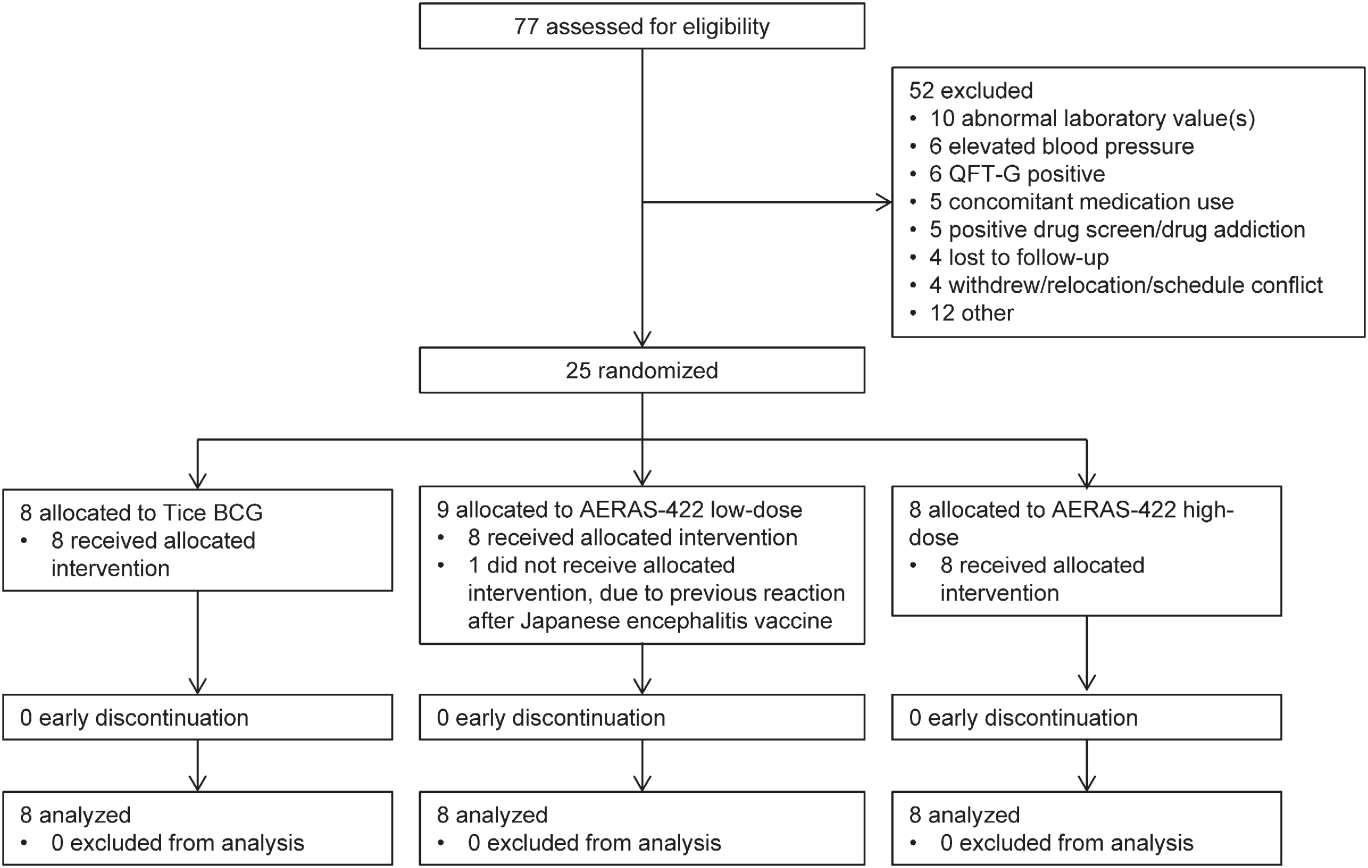
Consort Diagram.

Between Dec 06, 2010 and Aug 05, 2010, 24 volunteers were enrolled (AERAS-422 high dose, *n* = 8; AERAS-422 low dose, *n* = 8; Tice BCG, *n* = 8); all were included in the safety and immunogenicity analyses. All 24 randomized subjects were vaccinated on day 0 and completed the 182-day trial (Consort Diagram, Fig. 1). There were no major differences in demography between the groups (Supplemental Table 1). All subjects had at least one adverse event (AE; Supplemental Table 2). Adverse events primarily involved the expected local reactions known to occur with intradermal BCG vaccination. There were no clinically significant differences in these AEs between the groups (see Supplementary materials for further details).

### 3.2 Unexpected Safety Signal

**Fig. 2:**
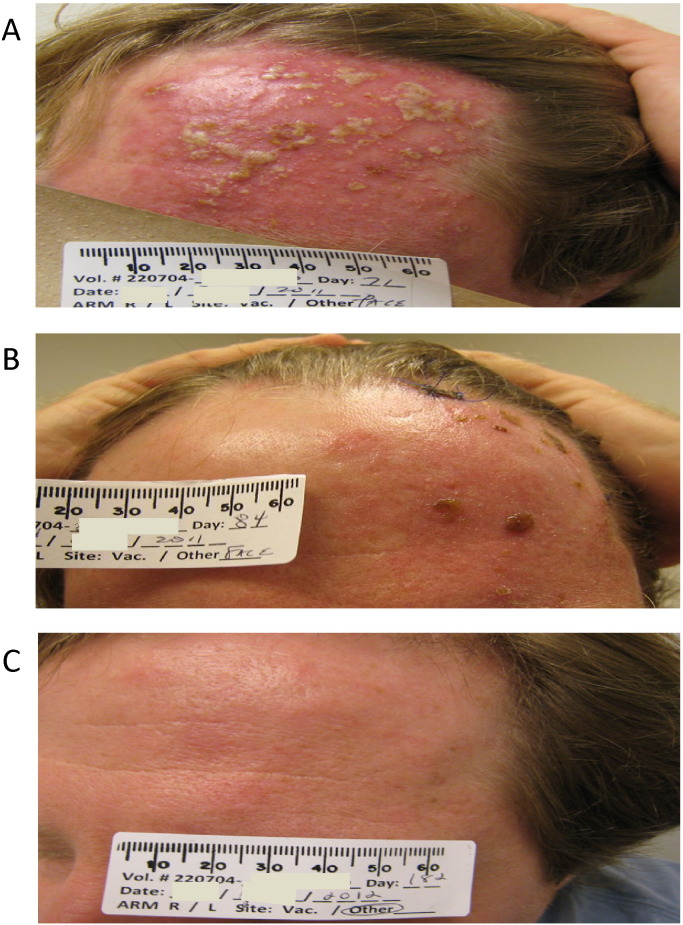
Subject with VZV reactivation 2 months after AERAS-422 vaccination. Shown are pictures of the 38 yo male who presented with a rash located on the left scalp and forehead, bilateral sinus congestion, sinus pain on the left, and diffuse headache described as aching and pressure 64 days after receiving AERAS-422. Examination of the skin lesion on the face (left side of the scalp to forehead) revealed a vesicular rash with pustules and scabs on an erythematous base in the CN5 V1 dermatome (A). There was also erythema of the left cornea, per examination by an ophthalmologist, as well as an erythematous and edematous left eyelid (data not shown). After zoster diagnosis and treatment, the subject was seen on day 84, when the condition had noticeably improved (B). The subject completed the study through the day 182 evaluation, at which time the herpes zoster reactivation was resolved with no sequelae (C).

Unexpectedly, VZV reactivation (zoster) occurred in two of eight volunteers in the AERAS-422 high dose group. One volunteer, a previously healthy 38 year old Caucasian male, reported to the study clinic 64 days after vaccination with a painful rash over his left scalp and forehead, sinus congestion, and diffuse headache (Fig. 2). A second volunteer (a 21 year old Caucasian male) presented to an urgent care center 86 days post-vaccination with a mild to moderate painful vesicular rash over the posterior left shoulder (data not shown). These volunteers were treated with acyclovir or famciclovir, steroids (first volunteer only), and pain medication. All signs and symptoms resolved over 2 months without residual pain or other sequela for the first volunteer and within 22 days for the second volunteer (except for hypopigmented spots over the VZV-presenting area). A clinical diagnosis of zoster was made for both volunteers, and confirmed by significant increases in VZV-specific antibody titers (1:16 pre-vaccination to 1:2048 post-zoster for the first volunteer; 1:2 pre-vaccination to 1:256 post-zoster for the second volunteer; determined by Quest Diagnostics). The diagnosis for the first volunteer was confirmed by PCR of punch biopsy material. PCR of punch biopsy tissue from the second volunteer, done > 1 week after symptom resolution, was negative for VZV.

When it was determined that the two volunteers with zoster had received AERAS-422, after unblinding was requested by the study Data Safety Monitoring Board, clinical development of AERAS-422 was discontinued. All 24 volunteers had serial VZV serology completed by a highly sensitive and specific indirect immunofluorescence assay (FAMA) to confirm the clinical diagnoses and rule out subclinical reactivation in any additional volunteers (Williams et al., 1974). No other subjects had increased VZV titers post-vaccination.

The remainder of this report focuses on investigations designed to explore the mechanism for zoster reactivation, as well as the expression of immune and gene patterns induced by the different vaccinations.

### 3.3 Antigen-specific T Cell Responses

**Fig. 3:**
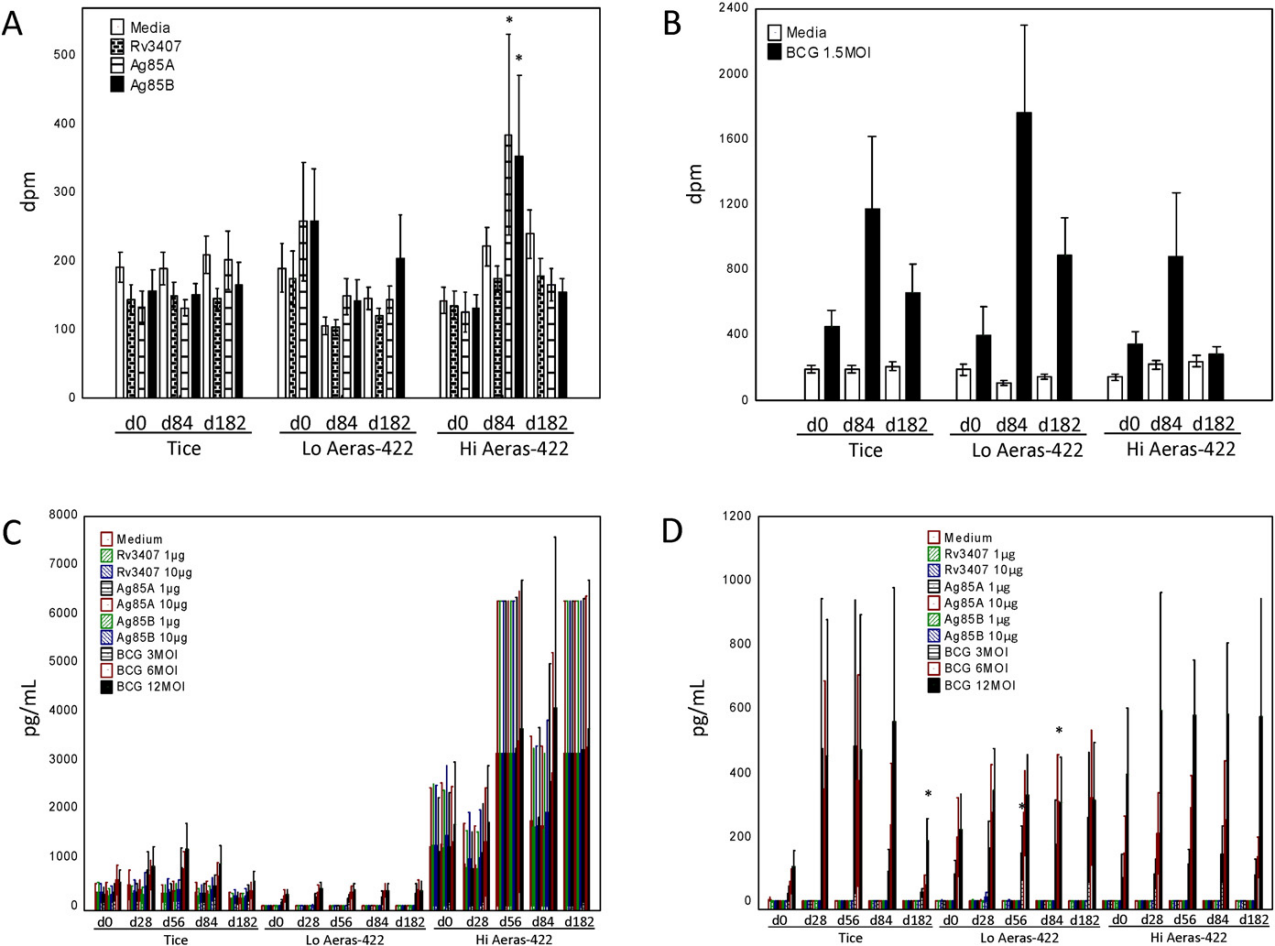
Vaccine-induced lymphoproliferative and IFN-γ responses induced by wild type, low dose AERAS-422 and high dose AERAS-422 BCG. Whole blood was collected pre- and post-vaccination and stimulated *in vitro* with different antigens (the recombinant proteins overexpressed by AERAS-422 and live Tice BCG). Antigen-specific lymphoproliferative responses were studied at days 0 (pre-vaccination), 84 (3 months post-vaccination) and 182 (6 months post-vaccination) (A and B). Antigen-specific IFN-γ responses stimulated *in vitro* with the recombinant proteins and different MOIs of Tice BCG at days 0, 28, 56, 84, and 182 are shown for all volunteers (C) and excluding the three volunteers with high spontaneous baseline responses (D). Mean and standard error responses are presented. *p < 0.05 by Wilcoxon matched pairs test.

Whole blood lymphoproliferation responses stimulated by overexpression of AERAS-422 recombinant antigens were studied in real-time during the trial. High dose AERAS-422 induced significant increases in both Ag85A and Ag85B responses 84 days post-vaccination (*p* < 0.05 compared with day 0 by Wilcoxon matched pairs testing); increases in Ag85A or Ag85B responses were not observed after Tice BCG or low dose AERAS-422 (Fig. 3A). No vaccinations were associated with induction of post-vaccination Rv3407-specific responses. Despite negative QuantiFERON TB Gold tests and no history of either known TB exposure or previous BCG vaccination, BCG *in vitro* stimulation induced substantial IFN-γ responses compared with medium and recombinant antigen stimulations in all groups, even prior to Tice or AERAS-422 vaccination. After *in vitro* restimulation with live wild type Tice BCG (Fig. 3B), all three vaccination groups developed similar increases in BCG-induced responses.

Whole blood IFN-γ secretion levels were measured as an estimate of overall vaccine-induced type 1 (CD4 + Th1 and CD8 + Tc1) immune responses after medium rest, recombinant antigen stimulation at two separate antigen concentrations, and BCG at three different MOIs stimulation (Fig. 3C). Significantly increased IFN-γ responses post-vaccination were not detected after stimulation with each of the overexpressed recombinant antigens or BCG in any vaccine group. However, three volunteers, (one in the high dose AERAS-422 group, two in the Tice group), produced > 10.000 pg/ml of IFN-γ even in the rested condition (data not shown).

When these three volunteers were excluded from the analysis, none of the vaccines induced detectable increases in IFN-γ following stimulation with the three recombinant antigens (Fig. 3D). In contrast, all three vaccine groups demonstrated similar post-vaccination increases in IFN-γ in response to restimulation with BCG, some of which were significantly increased compared with pre-vaccination (Wilcoxon matched pairs testing, *p* < 0.05). The highest post-vaccination BCG-specific responses were observed in the high dose AERAS-422 group, however, the overall responses for this group were not significantly higher than in the other two groups.

**Fig. 4:**
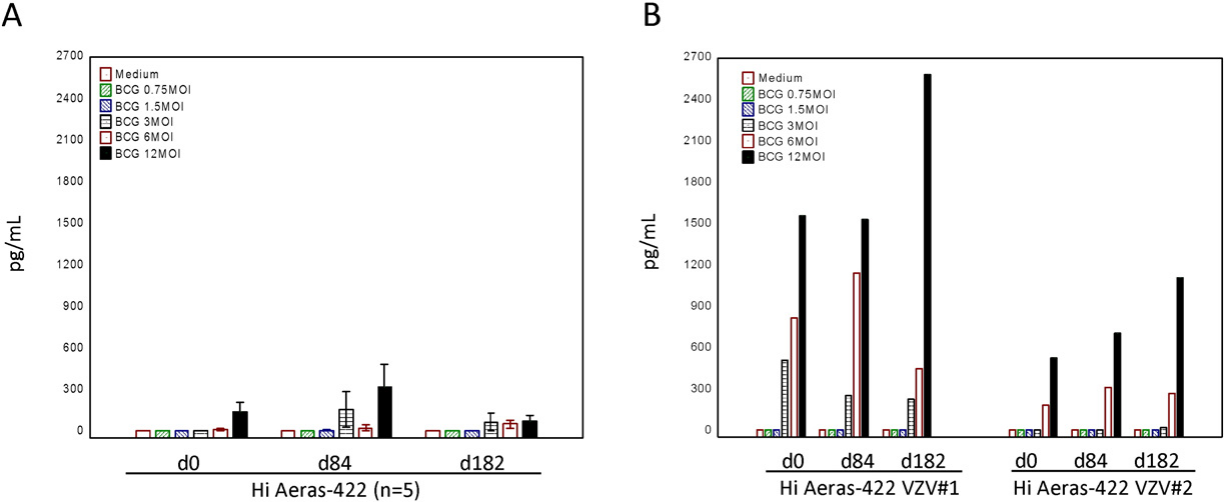
VZV reactivation was associated with exaggerated BCG-specific IFN-γ responses. Mean (with standard error) BCG-specific IFN-γ responses detected in the five high dose AERAS-422 volunteers that did not develop VZV reactivation (also excluding the one high spontaneous IFN-γ producer) are shown (A). BCG-specific IFN-γ results for the two volunteers in the high dose AERAS-422 group that developed zoster are also shown(B).

The two volunteers who suffered zoster post-vaccination displayed five-ten fold higher responses pre-vaccination after *in vitro* restimulation with BCG (Fig. 4B) compared with the other volunteers (excluding the high spontaneous IFN-γ producer) in the high dose AERAS-422 group (Fig. 4A), responses that were further augmented by AERAS-422 vaccination.

Intracellular cytokine responses (ICS) in frozen PBMC were also studied (Supplemental Fig. 1). No statistically significant increases in cytokine response were seen post-vaccination after restimulation with Rv3407, Ag85A, or Ag85B in any of the three treatment groups (see Supplementary materials for further details).

### 3.4 Inflammatory Responses in Human Monocytes

**Fig. 5:**
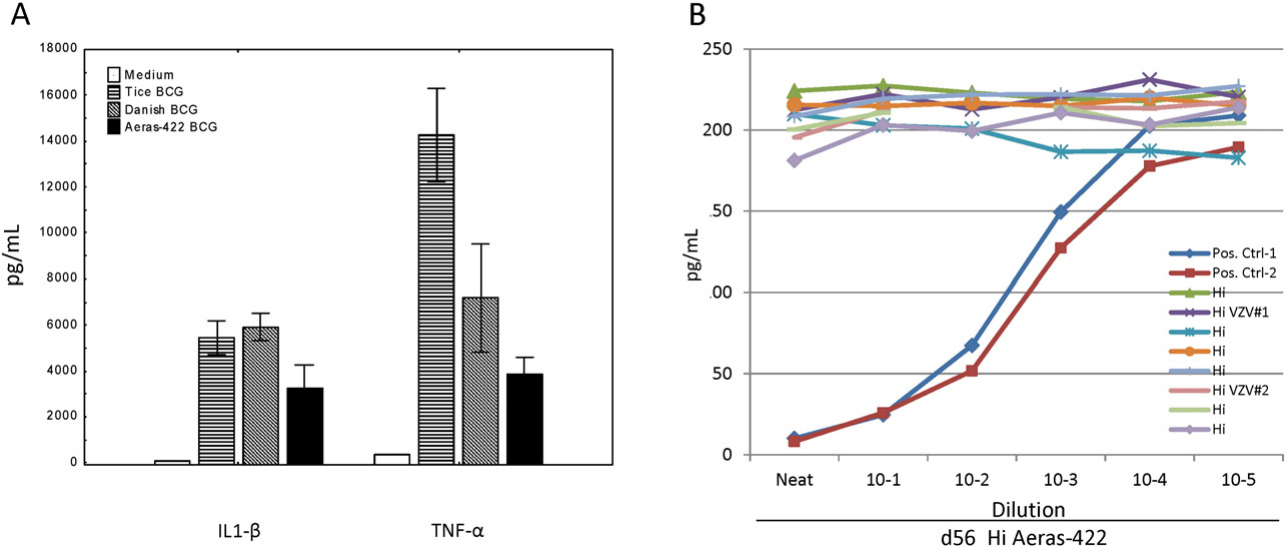
VZV reactivations were not related to increased monocyte inflammatory responses alone induced by AERAS-422 or to *anti*-IFN-γ-specific autoimmunity. IL-1β and TNF-α inflammatory cytokine responses from ELISAs of supernatants from human monocyte cultures infected for 72 h with wild type (Tice or Danish) or AERAS-422 recombinant BCG strains (MOI 30) are shown as mean and standard error (A). Results of IFN-γ neutralization assays using plasma samples harvested on day 56 from all eight high dose AERAS-422 recipients are shown as pg/ml for dilutions up to 10^− 5^ (B).

To address whether AERAS-422 induces a more robust innate immune cell inflammatory cytokine response than wild type BCG strains, human monocytes were infected with one of two strains of wild type BCG (Tice and Danish) or AERAS-422 and the production of secreted IL-1β and TNF-α measured. AERAS-422 did not induce exaggerated inflammatory cytokine responses, even after 72 h of infection with a wide range of MOI up to a maximum of 30 CFU/monocyte for each BCG strain (Fig. 5A).

### 3.5 Potential Involvement of IFN-γ-specific Autoimmunity in VZV Reactivation

To explore a genetic basis for predisposition to AERAS-422-induced zoster, HLA typing was completed for all subjects (data not shown). One of the volunteers who developed zoster was determined to express DRB1 16:01, very similar to the DRB1 16:02 allele previously associated with an autoimmune response targeting IFN-γ with neutralizing autoantibodies (Kampitak et al., 2011, Browne et al., 2012, Chi et al., 2013). The other zoster-positive volunteer exhibited DQB1 05:01 allele expression, closely related to the DQB1 05:02 allele, also associated with IFN-γ autoimmunity.

To test whether the exaggerated IFN-γ responses observed in both individuals with zoster were related to an autoimmune response leading to enhanced neutralization of IFN-γ biologic activity, plasma from all volunteers in the high dose AERAS-422 group was tested for the presence of IFN-γ neutralizing activity. Plasma harvested on day 56 (Fig. 5B), pre-vaccination and on day 84 (data not shown) did not display IFN-γ neutralization activity. Two volunteers in the high dose AERAS-422 group with no VZV reactivation also were determined to possess the DQB1 05:01 allele.

### 3.6 Whole Blood Bactericidal Activity Against Mycobacteria

**Fig. 6:**
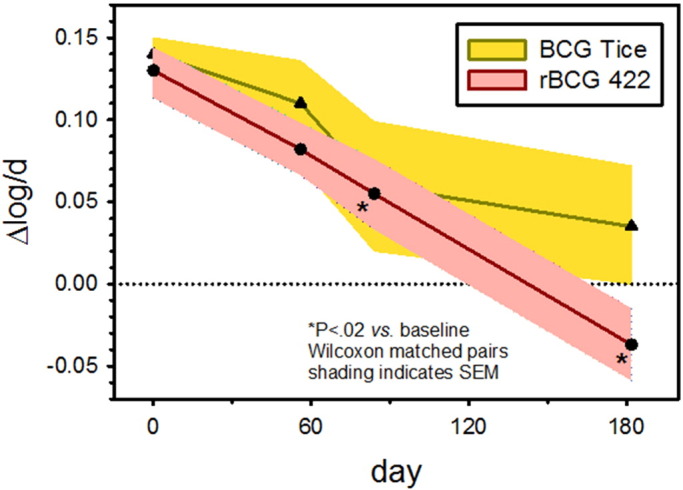
Whole blood bactericidal activity. The *anti*-mycobacterial activity of whole blood was studied using *M. bovis* BCG cultured with blood samples harvested from volunteers in the Tice and high dose AERAS-422 groups on days 0, 56, 84 and 182. The vertical axis indicates log change mycobacterial viability per day of whole blood culture, with positive numbers indicating growth. *p < 0.02 by Wilcoxon Matched-Pairs Test comparing pre- to post-vaccination responses.

Pre-vaccination, whole blood from subjects in both the Tice and AERAS-422 arms allowed 0.5 log BCG growth over 4 days (0.13 log/day). No statistically significant changes in WBA from baseline emerged in the Tice BCG group (Fig. 6), or the low dose AERAS-422 group (data not shown). In the high dose AERAS-422 group, statistically significant inhibition of growth compared to baseline was apparent by day 84, and significant bactericidal activity persisted on day 182 (Fig. 6). Any positive delta log/d result indicates net growth of BCG during the whole blood/BCG co-culture period. The mean delta log/day value of − 0.035 for the high dose AERAS-422 group on day 182 means the immune responses in whole blood could kill mycobacteria. This has never been seen before in previous TB vaccine trials. The two volunteers who developed zoster did not exhibit distinctly high or low WBA responses compared to other volunteers receiving high dose AERAS-422.

### 3.7 Myeloid Inflammatory Immune Responses

RNA sequencing of whole PBMC harvested pre- and post-vaccination was performed (Supplemental Fig. 2). In contrast to our previous work analyzing vaccine-induced innate immune responses that revealed differential expression of over 1000 genes (Zak et al., 2012), only weak responses were observed after BCG and rBCG vaccination (see Supplementary materials for further details).

### 3.8 Associations Between Transcriptomes and Vaccine-induced Mycobacterial Growth Inhibition

WBA responses induced by AERAS-422 were integrated with the PBMC transcriptomes. Longitudinal correlations between gene expression and WBA were evaluated at matching time points for matched trial participants. Unexpectedly, across all vaccine groups and time points, positive correlations were observed between both IL-1β and a monocyte chemokine module and delta WBA (Supplemental Fig. 3, Supplemental Fig. 4), indicating that decreased mycobacterial killing was associated with increased expression of myeloid associated and pro-inflammatory genes.

**Fig. 7:**
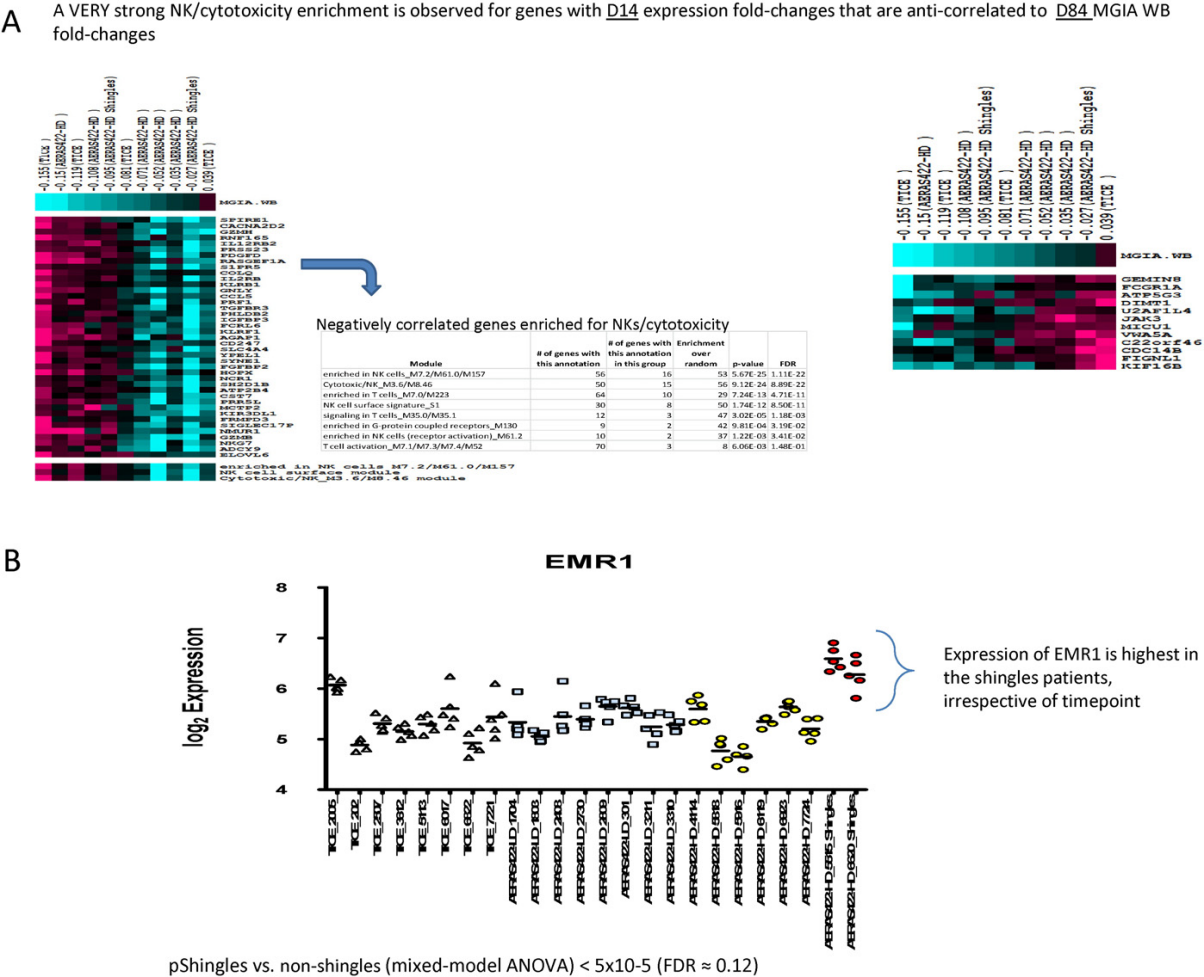
Correlations between post-vaccination transcriptomal signatures, BCG immunogenicity and VZV reactivation. Exploratory hypothesis-generating analyses were performed that integrated the transcriptome data with WBA and VZV reactivation datasets (A). Expression of EMR1 is shown for individual volunteers (B).

Gene expression and WBA were integrated at the level of vaccine-induced responses. Log2 fold-changes compared to pre-vaccination for gene expression were directly correlated with those for WBA, at the same or later time points. This analysis identified many genes with strong (*p* < 0.005) negative Spearman rank correlations between day 14 expression fold-changes and day 84 WBA changes (Fig. 7A). Module enrichment analysis of these genes revealed remarkably strong associations with NK cells, T cells, and cytotoxicity (four modules with FDR < 1 × 10^− 10^). Included among these genes was IL-12RB2, with a day 14 gene expression fold-change that robustly correlated with the day 84 WBA fold-change (*r* = − 0.9, *p* = 4 × 10^− 4^) (Supplemental Fig. 5). These results suggest that development of robust vaccine-induced mycobacterial killing at late time points is associated with robust induction of NK and cytotoxicity modules in PBMCs at earlier time points.

### 3.9 Gene Expression Changes Associated With VZV Reactivation

Targeted evaluations were performed to identify gene expression patterns preferentially associated with the two volunteers that developed zoster. Despite implementing numerous analytical strategies, no statistically significant or otherwise compelling associations between VZV reactivation and AERAS-422-induced gene expression responses were identified. The only robust association between gene expression and VZV reactivation observed was that both volunteers exhibited remarkably higher expression of the macrophage marker *F*4/80-encoding gene EMR1, irrespective of the time point analyzed and compared to all other volunteers in the trial (*p* < 5 × 10^− 5^; FDR ~ 12% by exhaustive permutation test that only evaluated the subset of genes that were as abundantly expressed and variable as EMR1; FDR = 100% without pre-filtering for abundance and variability; Fig. 7B).

## 4 Discussion

In this first-in-human phase I trial, two of eight volunteers administered the higher dose of AERAS-422 developed VZV reactivations 2–3 months post-vaccination. VZV reactivation has not previously been described as a complication of BCG vaccination. Based on the known epidemiology of VZV and reactivation, the chance that both cases were random events was estimated to be less than one in 200,000, strongly indicating some unique feature of the vaccine and/or of the immune response(s) induced by AERAS-422 was responsible. Hypotheses that could explain AERAS-422 induced VZV reactivation include reaction to intrinsic properties of PFO_G137Q_, effects from over-expression of *Mtb* antigens, or an imbalance of type I *vs.* type II interferon responses.

In the present study, increases in perfringolysin-specific antibodies were not identified post-vaccination in the serum of volunteers given AERAS-422 (data not shown), suggesting that the expressed levels of PFO_G137Q_ were too low *in vivo* to be recognized by the immune system. Therefore, we believe it is unlikely that the PFO_G137Q_ molecule expressed by AERAS-422 is responsible for the VZV reactivations. No associations between the expression of several molecules involved in immune-mediated cytotoxic activity and the VZV reactivations were found.

It is unlikely that overexpression of Ag85A and/or Ag85B was responsible for the VZV reactivations, as extensive pre-clinical and clinical use of these antigens in other experimental TB vaccines has been completed without reports of zoster. Overexpression of the Rv3407 antigen has not been previously studied in any other experimental TB vaccine clinical trial. However, we were unable to detect evidence of Rv3407-specific immunity induced by AERAS-422 (data not shown), suggesting only low levels of the antigen were produced *in vivo*.

Although type I IFN (IFN-α/IFN-β) induces important *anti*-viral effects and type II IFN exerts immunogenic activity against intracellular pathogens, both type I and type II IFNs appear to have counter-regulatory activities. Type I IFN can exacerbate *Mtb* infections in mice associated with reduced CD4 + Th1 cell responses (Manca et al., 2001) and further impair the ability of human macrophages to control the growth of intracellular mycobacteria (Bouchonnet et al., 2002, Teles et al., 2013). The molecular signature of active TB disease has been characterized as potent type I IFN signaling in human neutrophils (Berry et al., 2010) and demonstrated to reduce type II IFNR expression at the molecular level (Rayamajhi et al., 2010).

Pre-vaccination IFN-γ responses stimulated by BCG *in vitro* culture with PBMC, as demonstrated in all three vaccine groups in our study (Fig. 3B), have been seen previously and interpreted as either innate immune responses induced by BCG in NK cells or other nonspecific cells, or background memory T cell immunity directed against cross-reactive antigens expressed by environmental organisms other than *Mtb* (Hoft et al., 2002, Kemp et al., 1996, Hoft et al., 1998, Hoft et al., 1999, Hoft et al., 1999, Hoft et al., 2008). Although increases in BCG-induced responses in all three groups post-vaccination were not statistically significant (likely due to the high variability of responses and small sample size), these innate or cross-reactive background responses may be as important for predisposing subjects to an immunopathologic event such as VZV reactivation as any vaccine-induced immune responses.

A new *anti*-IFN-γ-specific autoimmune phenomenon has been identified that is associated with expression of certain HLA-DR/DQ alleles during dissemination of both mycobacterial infection and VZV reactivation (Kampitak et al., 2011, Browne et al., 2012, Chi et al., 2013), suggesting a role for type II IFN responses in suppression of *Mtb* replication and VZV reactivation. Combined with the high level BCG-induced IFN-γ responses detected in the two volunteers who developed zoster, these data engendered the possibility that these volunteers may possess *anti*-IFN-γ antibodies that neutralized cytokine function. Such an autoimmune response could lead to exaggerated IFN-γ responses trying to compensate for neutralizing antibody activity. The two zoster-presenting volunteers expressed HLA class II alleles previously associated with *anti*-IFN-γ autoimmunity, but so did other volunteers that did not develop zoster. Also, IFN-γ neutralizing activity was not detected in plasma harvested either pre- or post-vaccination from the volunteers who developed zoster, indicating that *anti*-IFN-γ autoimmunity was unlikely to be responsible for a defect in IFN-γ effector function. Despite the lack of detected autoimmunity, the similarity of the Class II HLA alleles between the two zoster-presenting volunteers, combined with complete absence of similar Class I alleles, suggests an association of VZV reactivation with Class II. The mechanisms of this potential association are not understood.

Whether too much type II IFN may inhibit type I IFN responses involved in maintenance of VZV latency is unknown. However, both volunteers who developed zoster displayed hypersensitive IFN-γ responses after whole blood stimulation with BCG *in vitro* pre- and post-vaccination, responses that were five-ten fold higher than those observed in other high dose AERAS-422 recipients pre-vaccination and that increased post-vaccination. Future research should investigate negative effects of type II IFN on type I IFN signaling and functional effects.

No vaccine-induced immune responses were associated with the zoster events either in targeted immune or whole genome expression studies. This could be due to the absence of appropriate temporal sampling for the transcriptome analyses (no measurements were taken before 14 days post-vaccination), or because the processes that led to VZV reactivation are not related to systemic inflammatory responses that would be measurable in peripheral blood transcriptomes. Both volunteers who developed zoster were found to constitutively express higher levels of EMR1 in their PBMCs. *F*4/80, the mature protein product of EMR1, has been identified on murine microglial cells (Zhou et al., 2013), and EMR1 has been shown to be expressed by human eosinophils (Legrand et al., 2014, Hamann et al., 2007), both of which could be relevant for control of VZV in latently infected individuals. The significance of high expression of EMR1 in the two zoster volunteers is unclear, but could represent high myeloid activation even at baseline, perhaps in some way influencing cross-talk between T cells and myeloid cells. Up-regulation of IFN-γ responses by AERAS-422 potentially could have led to higher suppression of type I IFN control of VZV reactivation. An *in vitro* guinea pig model for studies of VZV latency and reactivation has been developed to evaluate whether high doses of IFN-γ plus/minus activated myeloid cells (especially eosinophils) can increase VZV reactivation (Chen et al., 2011, Gan et al., 2014).

In spite of the absence of robust associations between the transcriptomes and VZV reactivation, compelling and functionally relevant vaccine-induced transcriptome changes were observed that involved differential regulation of genes associated with T cell, myeloid cell, and inflammatory modules. Interestingly, up-regulation of the myeloid module-associated genes temporally matched the known peak of *in vivo* BCG replication.

In this trial, an earlier and more robust activation of NK/cytotoxic responses was correlated with increased capacity of whole blood cell populations to inhibit mycobacterial growth post-vaccination (Fig. 7A). Control of mycobacterial growth is an important goal of the protective immune response against disseminated TB disease, and therefore these results suggest that early activation of NK/cytotoxic activity may serve as an important new target for TB vaccine improvement. In contrast, monocyte inflammatory responses were found to be negatively associated with mycobacterial growth inhibitory activity. This could be due to increased myeloid cell numbers providing a larger reservoir of target cells for infection, and/or direct interference of these cells with mycobacterial growth inhibitory mechanisms. Combined, these correlation analyses suggest that post-vaccination increases in mycobacterial growth inhibitory activity may be a manifestation of adaptive immune responses that can be prevented by too much myeloid inflammation. Additional work to confirm the importance of these early responses for optimal induction of protective long-term memory and effector functions against TB is warranted, as activators of the correct early innate immune responses could possess ideal adjuvant properties for subunit or other vaccines.

Overall, these results suggest that the combination of an exaggerated IFN-γ response and a myeloid activation state, resulting from vaccination with this rBCG vaccine, may have triggered an unexpected VZV reactivation in two young and otherwise healthy male participants in this Phase 1 study, necessitating the discontinuation of the development of this vaccine.

The following are the supplementary data related to this article.

Supplementary material. Image 1

Supplemental Fig. 1 Total ICS responses. CD4 + (A and B) and CD8 + (C and D) responses following stimulation with Ag85A (A and C) or Ag85B (B and D) are shown. Boxes extend from the 25th to the 75th percentile. Bars within boxes represent median response for each group. The “X” represents the mean response for each group. Whiskers represent the min and max. Data are shown for Tice BCG (black box plots), AERAS-422 low dose (red box plots) and AERAS-422 high dose (blue box plots) groups. Supplemental Fig. 1

Supplemental Fig. 2 Post-vaccination changes in whole genome-wide transcriptomal responses. Total RNA from whole PBMC harvested from days 0, 14, 28, 56 and 84 was analyzed by RNAseq. Shown are heat maps for the significantly altered gene sets that are over-represented for associations with specific blood transcriptional modules (A: T cell proliferation module; B: monocyte modules; C: T cell and AP-1 modules). Pink gene up-regulation; blue: down-regulation (compared to pre-vaccination baseline). Supplemental Fig. 2

Supplemental Fig. 3 Increases in IL-1β expression are anti-correlated with mycobacterial growth inhibitory activity. IL-1β mRNA expression was positively correlated with delta log growth/day (i.e. – more IL-1β expression led to less mycobacterial killing) across all vaccines and all time points. Supplemental Fig. 3

Supplemental Fig. 4 Myeloid chemokine expression is anti-correlated with mycobacterial growth inhibitory activity. Genes associated with a previously identified myeloid chemokine module were observed to be positively correlated with delta log growth/day (i.e. – more myeloid chemokine expression led to less mycobacterial killing) across all vaccines and all time points. Supplemental Fig. 4

Supplemental Fig. 5 IL-12RB2 induction is associated with enhanced whole blood mycobacterial growth inhibitory activity. Day 14 fold changes for IL-12RB2 were inversely correlated with day 84 Delta log growth/day (r = − 0.9, p = 4 × 10^− 4^) (i.e. - more IL12RB2 expression led to more mycobacterial killing). Supplemental Fig. 5

Supplemental Table 1 Demographics. Supplemental Table 1

Supplemental Table 2 Adverse events in ≥ 2 subjects receiving AERAS-422. Supplemental Table 2

## Contributors

DFH, AB, AS, AT, JT, and GA contributed to the implementation of the study and supervision at the study site. DFH was the principal investigator. RW, BM, and DAH contributed to the study design. All authors (including BS, a medical writer at Aeras) contributed to data analysis and contributed to the writing and approval of the report.

## Declaration of interests

We declare no competing interests.
