# Supplementary material for: Safety and Immunogenicity of the Recombinant BCG Vaccine AERAS-422 in Healthy BCG-naïve Adults: A Randomized, Active-controlled, First-in-human Phase 1 Trial
Source: eBioMedicine. 2016 Apr 19;7:278–86. doi: 10.1016/j.ebiom.2016.04.010 (PMC4909487; doi:10.1016/j.ebiom.2016.04.010)
Supplement: Supplementary file 1 — Supplementary material. [file mmc1.docx]

**Supplementary Materials**

Follow up and Safety Evaluations

Vital signs (blood pressure, pulse, and temperature) were measured at screening, before vaccination and at 30 and 60 minutes after vaccination, and at all subsequent study visits through the end of the study. Chemistry, hematology, and urinalysis safety parameters were assessed from samples collected at screening and days 7, 14, 28, 56, 84, 112, and 140. PT and PTT were also measured at these time points.

A trend for a dose-dependent lowering of blood fibrinogen levels in the AERAS-422 groups was observed (0/8 in the Tice group, 1/8 in the low dose AERAS-422 group and 4/8 in the high dose AERAS-422 group; Supplemental Table 2). However, there was no similar trend for prolonged prothrombin time in AERAS-422 recipients, no bleeding complications occurred during the trial.

**Intracellular cytokine staining assay (ICS) (7-color)**

The frequency and pattern of Ag-specific cytokine-producing T cells in the blood were determined. Frozen PBMCs were prepared at the approved clinical trial site in St. Louis, MO, USA. The specimens were collected on days 0, 14, 28, 56, 84, 112, 140, and 182. Peptides derived from *M. tuberculosis* antigens Ag85A, Ag85B, and Rv3407 were obtained from JPT Peptide Technologies, GmbH (Berlin, Germany) and reconstituted in DMSO at a concentration of 250 μg/peptide/ml and stored at -80°C. Peptides consisted of 15-mers overlapping by 11 amino acids and spanning the full length of the protein.

Shown in Supplemental Figure 1 are the percentages of CD4+ (A and B) and CD8+ (C and D) T cells expressing one or more of the cytokines studied (IFN-γ, TNF-α and IL-2) after restimulation with Ag85A (A and C) or Ag85B (B and D) peptide pools (data not shown for restimulation with Rv3407). Although some volunteers in the AERAS-422 groups developed increases in these responses post-vaccination, the post-vaccination increases in all groups did not achieve statistical significance. The failure to detect any significantly increased vaccine-induced responses, unlike in the whole blood studies, may be related to differences in sensitivity studying fresh whole blood vs frozen PBMC. However, these ICS results are consistent with the whole blood studies in that in neither type of assay did we detect significant increases in post-vaccination cytokine responses reactive with the recombinant antigens.

**RNA collection and processing**

Aliquots of 2 million PBMC collected on days 0, 14, 28, 56, 84 and 182 were suspended in TRIzol (Life Technologies, Inc.), and frozen. Total RNA was extracted following the manufacturer’s instructions.

**RNA-sequencing (RNA-Seq)**

cDNA library preparation and RNA sequencing were performed by Expression Analysis, Inc. (Durham, NC). cDNA library preparation was performed using Illumina (San Diego, CA) TruSeq Stranded Total RNA Sample Prep Kit according to the manufacturer’s instructions. The sequencing strategy was 40 million 50bp paired-end reads, and sequencing was performed on Illumina (San Diego, CA) HiSeq-2000 sequencers. FASTQ sequences were transferred to Seattle BioMed for analysis.

**QC and processing of RNA-Seq data**

Read pairs were preprocessed using in-house scripts that adjust base calls with phred scores < 5 to ‘N’ and remove read pairs for which either end has fewer than 30 unambiguous base calls, a method that also indirectly also removes pairs containing mostly adaptor sequences. The median depth of RNA sequencing after post-processing was 41 million read pairs. Read pairs were aligned to the human genome (hg19) using STAR (v2.3.1d),^1^ taking as input the Ensembl GRCh37.74 splice junction table but allowing novel splice junction detection. The mean percentage of preprocessed reads mapped was 96% and the mean GC content was 47%. Mapped read pairs were assigned to genes by collapsing all transcripts into a single gene model and then counting the number of reads that fully overlap the resulting exons using htseq (v. 0.6.0)^2^ with strict intersection and including strand information. Gene models for protein-coding genes were downloaded Ensembl (GRCh37.74). Reads that mapped to multiple locations were only counted once and those mapping to ambiguous regions were excluded. The mean percentage of mapped reads aligning to exonic regions of protein-coding genes was 55% while the intronic regions accounted for 22% of the mapped reads. Log2-transformed values of counts normalized by adjusted library counts were computed using the cpm function of the edgeR package.^3^ All subsequent statistical analyses of the RNA-Seq data were performed using the Log2 normalized gene-level read count data. Raw RNA-Seq data (FASTQ files) and normalized read count data will be deposited to the Gene Expression Omnibus.

**Statistical RNA-Seq analyses- identifying genes differentially expressed in response to vaccination**

Mixed-model analysis of variance (MMANOVA) as implemented in the R package LME4 was employed to identify genes with expression levels that varied significantly upon vaccination. Trial participant ID was used as the random effect in the models. Prior to statistical testing, the bottom 5% of genes in terms of expression signal and variation were excluded. Significant vaccine effects were identified by Chi-square test comparing a MMANOVA model with a fixed effect for time to a MMANOVA model without the time effect. Trial participants from all vaccine groups were analyzed together. P-values were adjusted for multiple comparisons using the Benjamini-Hochberg algorithm. Fifty-three genes with weakly statistically significant vaccine-induced responses were identified after filtering (p<0·005, false discovery rate [FDR]<0·5, and significant paired t-test for at least one vaccine for at least one time point).

**Statistical analyses integrating RNA-Seq and WBA**

Two exploratory analyses were performed to link RNA-Seq measurements with WBA. The first analysis directly compared expression levels with WB-MGIA data for matching participants at matching time points. Genes or modules with significant (p<0·01) Pearson correlations were retained. The second analysis compared expression responses (log2 fold-change compared to pre-vaccination) to WBA responses (log2 fold-change compared to pre-vaccination) at the same time point or at a later time point. Genes with significant Spearman correlations (p<0·005) were retained. The more stringent significance threshold was applied in the latter analysis given that a much smaller number of data points were available for computing the correlations.

**Statistical RNA-Seq analyses – identifying genes differentially expressed in participants that experienced VZV re-activation**

Mixed-model analysis of variance (MMANOVA) as implemented in the R package LME4 was employed to identify genes with expression levels that differed significantly between trial participants that experienced VZV reactivation and those that did not. Trial participant ID was used as the random effect in the models. Significant vaccine effects were identified by Chi-square test comparing a MMANOVA model with a fixed effect for VZV reactivation to a MMANOVA model without the VZV effect. Trial participants from all vaccine groups were analyzed together.

**Myeloid inflammatory immune responses**

RNA sequencing of whole PBMC harvested pre- and post-vaccination was performed. Only weak responses were observed after BCG and rBCG vaccination. This may be the result of the very late time points of RNA sampling in the present study. A small group of 23 genes associated with T cell proliferation was significantly up-regulated post-vaccination (Supplemental Figure 2A) for prolonged periods (≥84 days post-vaccination). This is consistent with the known persistence of BCG replication in vivo post-vaccination.^4^ A separate group of genes enriched for associations with monocyte and inflammatory response modules was also markedly elevated post-vaccination, although these responses peaked earlier (day 28-56) and returned to baseline by day 84 post-vaccination (Supplemental Figure 2B). Five modules were significantly associated with this group of genes (p<1·0x10^-3^; FDR<1·80x10^-1^), including modules associated with Toll-like Receptor (TLR) activation and inflammatory signaling, a monocyte surface signature, enrichment for monocytes, general inflammation and general monocyte specific genes. A third group of response genes was identified that was generally down-regulated post-vaccination. This gene set was enriched for associations with T cell signaling (p<8·3x10^-5^; FDR<1·8x10^-2^) and AP-1 transcription modules (p<2·8x10^-4^; FDR<3·0x10^-2^) (Supplemental Figure 2C).

**Supplementary Information References:**

1. Dobin A, Davis CA, Schlesinger F, et al. STAR: ultrafast universal RNA-seq aligner. *Bioinformatics* 2013; **29**: 15–21.

2. Anders S, Pyl PT, Huber W. HTSeq-a Python framework to work with high-throughput sequencing data. *Bioinformatics* 2015; **31**: 166–169.

3. McCarthy DJ, Chen Y, Smyth GK. Differential expression analysis of multifactor RNA-Seq experiments with respect to biological variation. *Nucleic Acids Res* 2012; **40**: 4288–4297.

4. Hoft DF, Leonardi C, Milligan T, et al. Clinical reactogenicity of intradermal Bacille Calmette-Guerin vaccination. *Clinical Infectious Diseases* 1999; **28**: 785–790.
