# Supplementary figures and images for: Safety and Immunogenicity of the Recombinant BCG Vaccine AERAS-422 in Healthy BCG-naïve Adults: A Randomized, Active-controlled, First-in-human Phase 1 Trial
Source: eBioMedicine. 2016 Apr 19;7:278–86. doi: 10.1016/j.ebiom.2016.04.010 (PMC4909487; doi:10.1016/j.ebiom.2016.04.010)

# Supplemental Figure #1

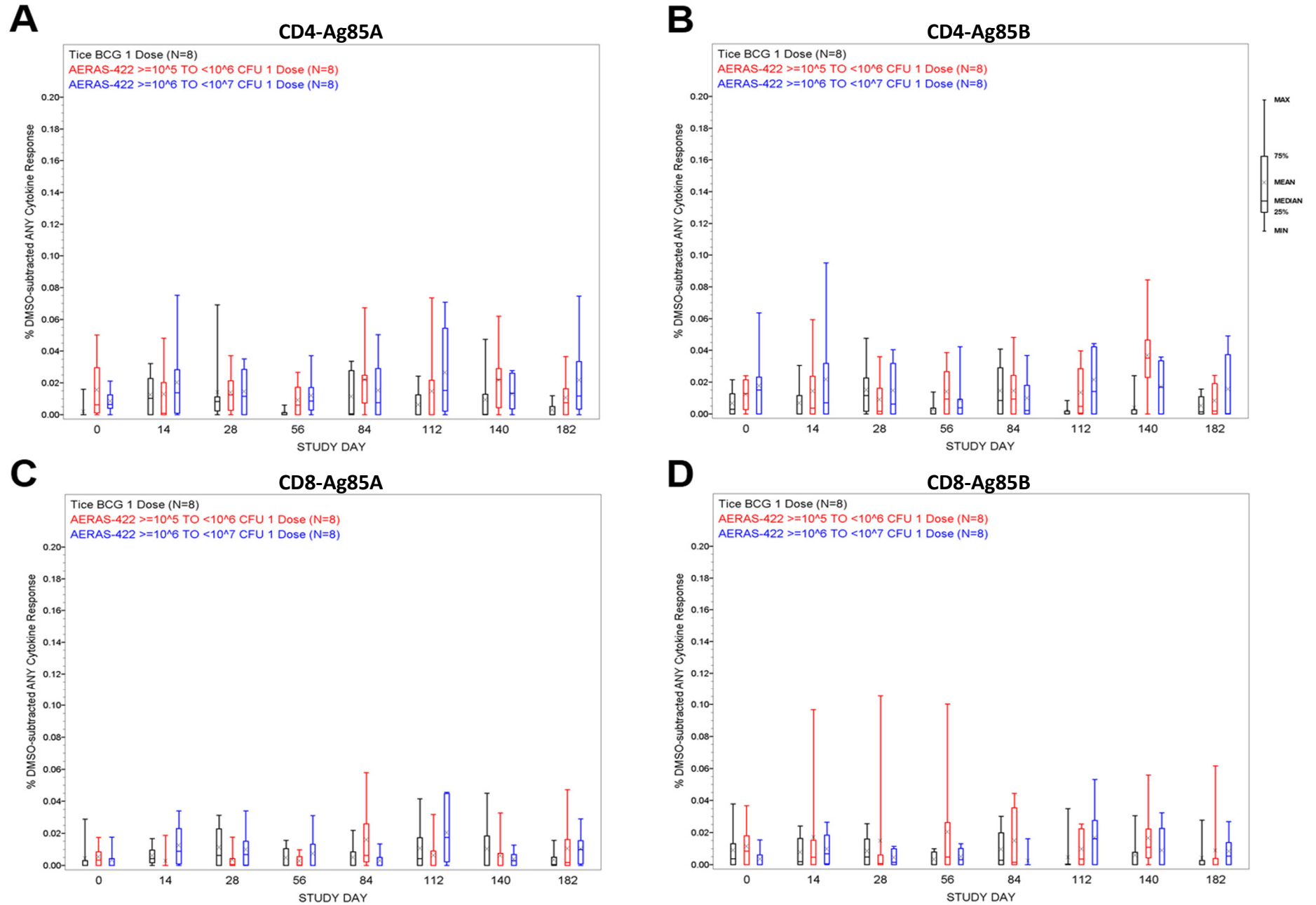

Supplement: Supplemental Fig. 1 — Total ICS responses. CD4 + (A and B) and CD8 + (C and D) responses following stimulation with Ag85A (A and C) or Ag85B (B and D) are shown. Boxes extend from the 25th to the 75th percentile. Bars within boxes represent median response for each group. The “X” represents the mean response for each group. Whiskers represent the min and max. Data are shown for Tice BCG (black box plots), AERAS-422 low dose (red box plots) and AERAS-422 high dose (blue box plots) groups. [file mmc2.pdf]

## Supplemental Figure #5

D14 FC for IL-12RB2 is anticorrelated with WBA FC at D84

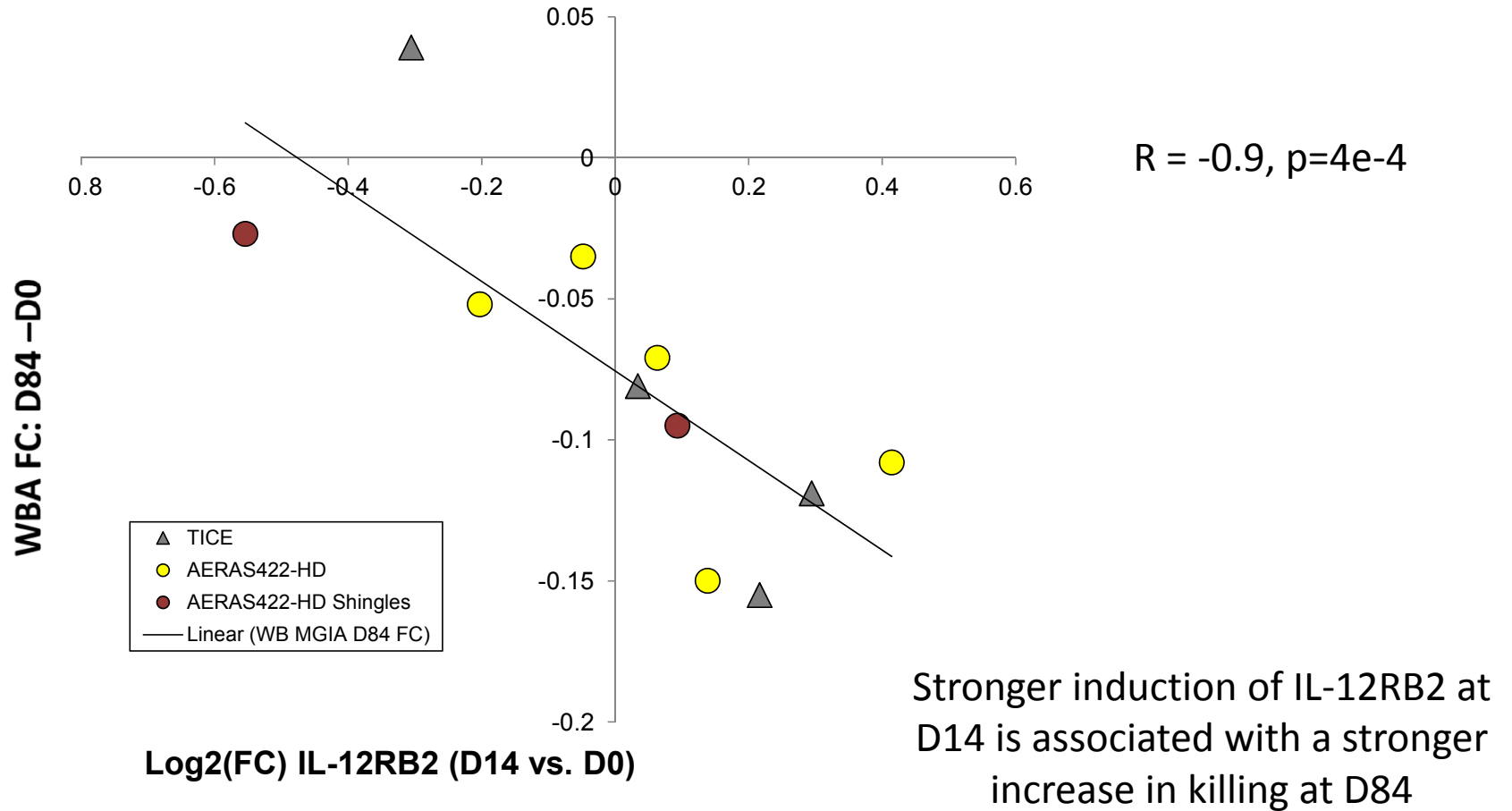

Supplement: Supplemental Fig. 5 — IL-12RB2 induction is associated with enhanced whole blood mycobacterial growth inhibitory activity. Day 14 fold changes for IL-12RB2 were inversely correlated with day 84 Delta log growth/day (r = − 0.9, p = 4 × 10− 4) (i.e. - more IL12RB2 expression led to more mycobacterial killing). [file mmc6.pdf]
