# Supplementary material for: Safety and Immunogenicity of the Recombinant BCG Vaccine AERAS-422 in Healthy BCG-naïve Adults: A Randomized, Active-controlled, First-in-human Phase 1 Trial
Source: eBioMedicine. 2016 Apr 19;7:278–86. doi: 10.1016/j.ebiom.2016.04.010 (PMC4909487; doi:10.1016/j.ebiom.2016.04.010)
Supplement: Supplemental Fig. 2 — Post-vaccination changes in whole genome-wide transcriptomal responses. Total RNA from whole PBMC harvested from days 0, 14, 28, 56 and 84 was analyzed by RNAseq. Shown are heat maps for the significantly altered gene sets that are over-represented for associations with specific blood transcriptional modules (A: T cell proliferation module; B: monocyte modules; C: T cell and AP-1 modules). Pink gene up-regulation; blue: down-regulation (compared to pre-vaccination baseline). [file mmc3.pdf]

Supplemental Figure #2

A

T cell proliferation module is enriched in genes showing diffuse up-regulation

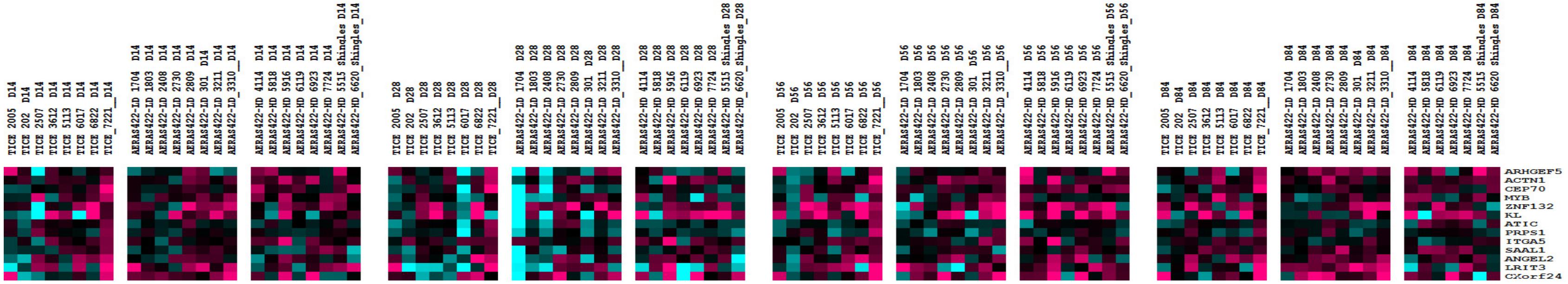

Module	# of genes with this annotation	# of genes with this annotation in this group	Enrichment over random	p-value	FDR
cell division stimulated CD4+ T cells_M46	23.0	2.0	76.8	2.95E-04	6.32E-02

B

Monocyte modules are enriched in genes that peak at D28-56

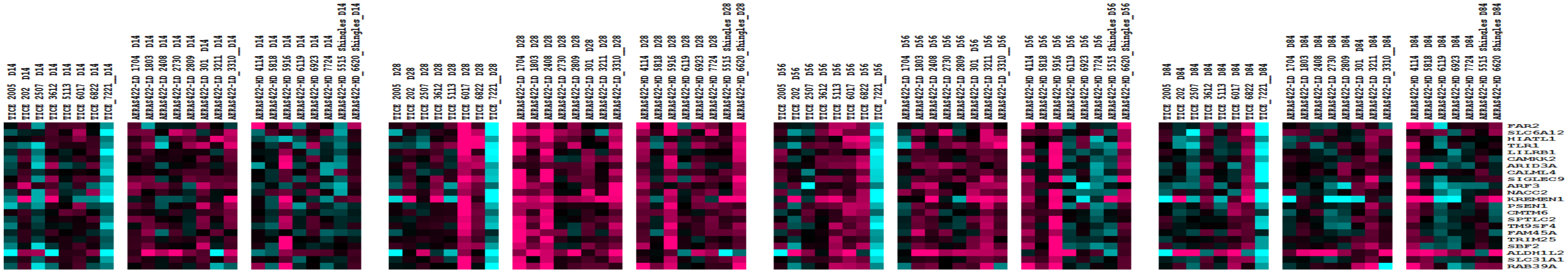

Module	# of genes with this annotation	# of genes with this annotation in this group	Enrichment over random	p-value	FDR
TLR and inflammatory signaling_M16	43.0	3.0	36.4	7.16E-05	1.53E-02
Monocyte surface signature_S4	90.0	3.0	17.4	6.43E-04	6.88E-02
enriched in monocytes_M4.15/M11.0/M73/M118.0	246.0	4.0	8.5	1.11E-03	7.90E-02
Inflammation_M3.2/M4.13/M4.2/M4.6/M5.1/M5.7/M7.1	711.0	6.0	4.4	1.74E-03	9.33E-02
Monocytes_M4.14	51.0	2.0	20.5	4.22E-03	1.80E-01

C

T cell and AP-1 modules are enriched in genes showing diffuse down-regulation

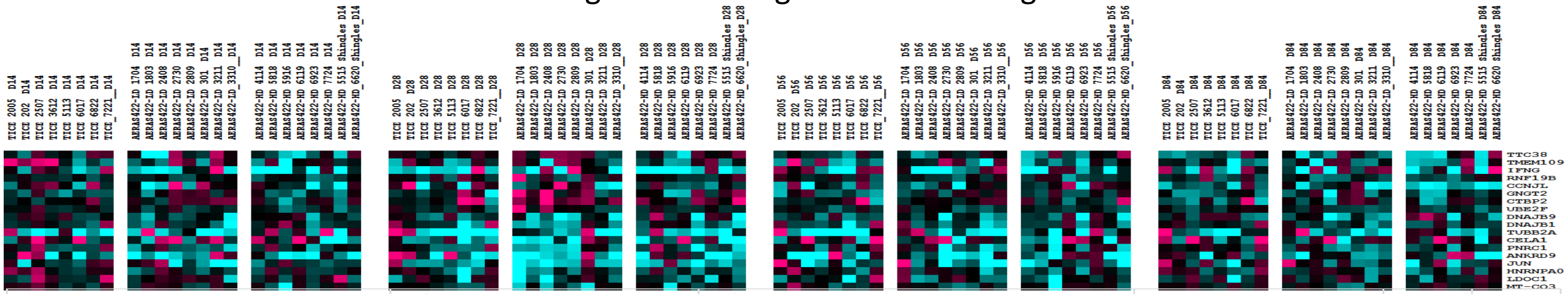

Module	# of genes with this annotation	# of genes with this annotation in this group	Enrichment over random	p-value	FDR
AP-1 transcription factor network_M20	9.0	2.0	141.8	8.29E-05	1.77E-02
signaling in T cells_M35.0/M35.1	16.0	2.0	79.8	2.75E-04	2.94E-02
