# Supplementary material for: Safety and Immunogenicity of the Recombinant BCG Vaccine AERAS-422 in Healthy BCG-naïve Adults: A Randomized, Active-controlled, First-in-human Phase 1 Trial
Source: eBioMedicine. 2016 Apr 19;7:278–86. doi: 10.1016/j.ebiom.2016.04.010 (PMC4909487; doi:10.1016/j.ebiom.2016.04.010)
Supplement: Supplemental Fig. 3 — Increases in IL-1β expression are anti-correlated with mycobacterial growth inhibitory activity. IL-1β mRNA expression was positively correlated with delta log growth/day (i.e. – more IL-1β expression led to less mycobacterial killing) across all vaccines and all time points. [file mmc4.pdf]

Supplemental Figure #3

IL1B (IL-1β) expression is positively correlated with WBA measurement, across vaccines and time points

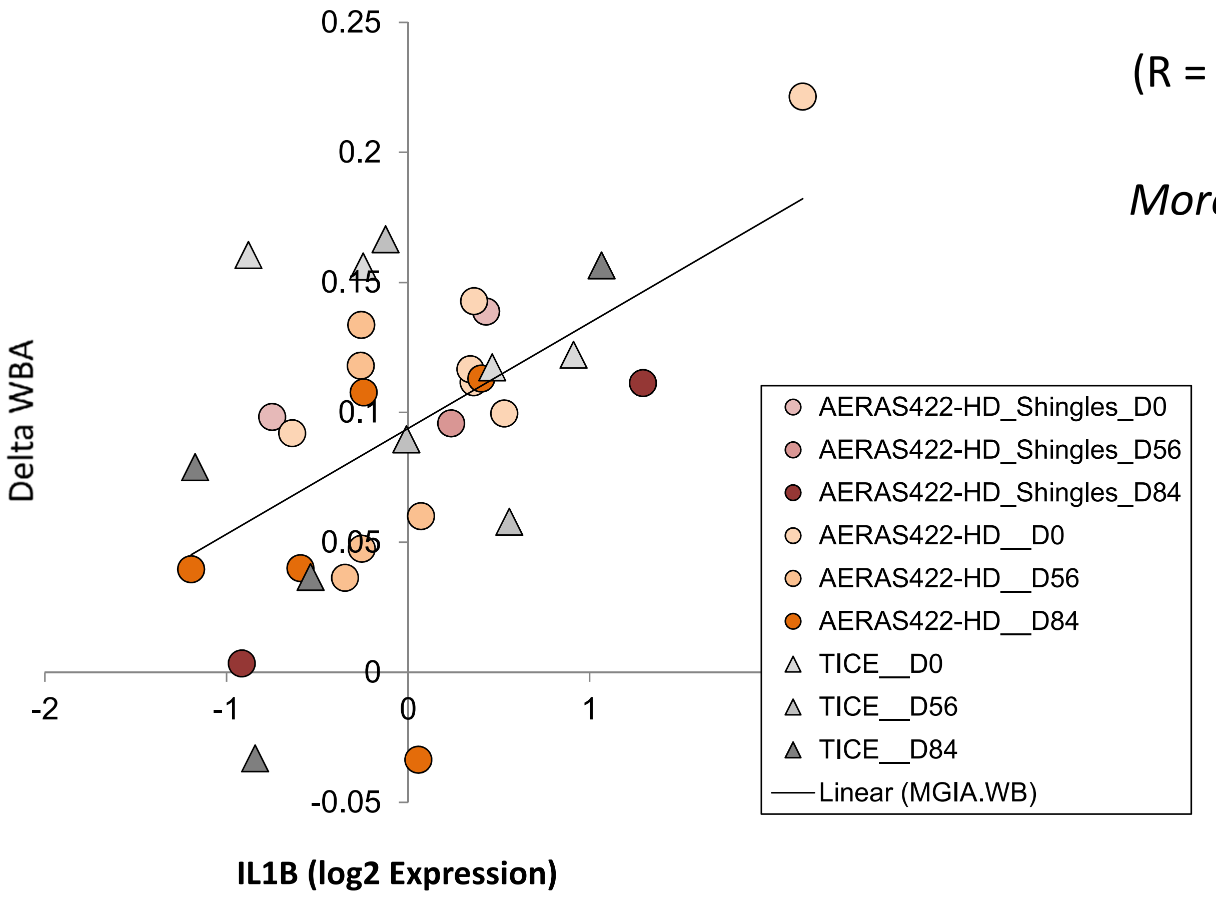

(R = 0.53, p = 0.002)

More IL-1β *corresponds to less killing*
