# Supplementary material for: Safety and Immunogenicity of the Recombinant BCG Vaccine AERAS-422 in Healthy BCG-naïve Adults: A Randomized, Active-controlled, First-in-human Phase 1 Trial
Source: eBioMedicine. 2016 Apr 19;7:278–86. doi: 10.1016/j.ebiom.2016.04.010 (PMC4909487; doi:10.1016/j.ebiom.2016.04.010)

Supplemental Figure #4

"Myeloid chemokine module" expression is positively correlated with WBA measurement, across vaccines and time points

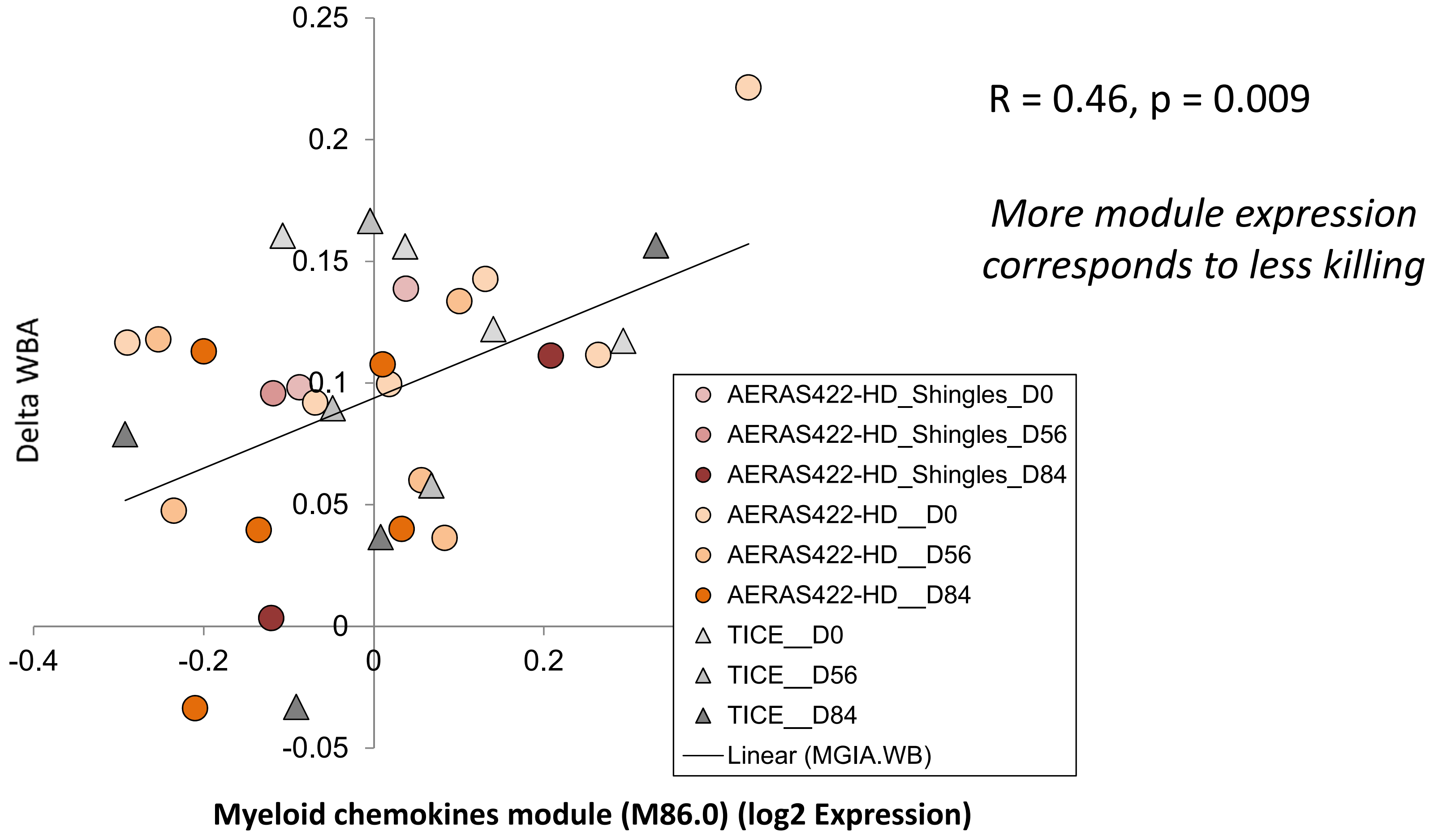

Supplement: Supplemental Fig. 4 — Myeloid chemokine expression is anti-correlated with mycobacterial growth inhibitory activity. Genes associated with a previously identified myeloid chemokine module were observed to be positively correlated with delta log growth/day (i.e. – more myeloid chemokine expression led to less mycobacterial killing) across all vaccines and all time points. [file mmc5.pdf]
