# Supplementary material for: Safety and Immunogenicity of the Recombinant BCG Vaccine AERAS-422 in Healthy BCG-naïve Adults: A Randomized, Active-controlled, First-in-human Phase 1 Trial
Source: eBioMedicine. 2016 Apr 19;7:278–86. doi: 10.1016/j.ebiom.2016.04.010 (PMC4909487; doi:10.1016/j.ebiom.2016.04.010)
Supplement: Supplemental Table 1 — Demographics. [file mmc7.docx]

**Supplemental Table 1. Demographics.**

| **Parameter** | **Tice BCG** | **AERAS-422** | | **Total**  **(N=24)** |
| --- | --- | --- | --- | --- |
|  |  | **Low-dose**  **≥10^5^ to <10^6^ CFU** | **High-dose**  **≥10^6^ to <10^7^ CFU** |  |
|  | **(N=8)** | **(N=8)** | **(N=8)** |  |
| Age (years) |  |  |  |  |
| Mean | 27.6 | 28.0 | 31.4 | 29.0 |
| SD | 7.27 | 5.24 | 7.58 | 6.70 |
| Gender, n(%) |  |  |  |  |
| Male | 4 ( 50.0) | 6 ( 75.0) | 4 ( 50.0) | 14 ( 58.3) |
| Race, n(%) |  |  |  |  |
| Black or African American | 0 ( 0.0) | 1 ( 12.5) | 0 ( 0.0) | 1 ( 4.2) |
| White | 7 ( 87.5) | 7 ( 87.5) | 8 (100.0) | 22 ( 91.7) |
| Other | 1 ( 12.5) | 0 ( 0.0) | 0 ( 0.0) | 1 ( 4.2) |
