# Supplementary material for: Safety and Immunogenicity of the Recombinant BCG Vaccine AERAS-422 in Healthy BCG-naïve Adults: A Randomized, Active-controlled, First-in-human Phase 1 Trial
Source: eBioMedicine. 2016 Apr 19;7:278–86. doi: 10.1016/j.ebiom.2016.04.010 (PMC4909487; doi:10.1016/j.ebiom.2016.04.010)
Supplement: Supplemental Table 2 — Adverse events in ≥ 2 subjects receiving AERAS-422. [file mmc8.docx]

**Supplemental Table 2. Adverse Events in ≥2 Subjects Receiving AERAS-422.**

| **Preferred Term** | **Tice BCG** | **AERAS-422** | |
| --- | --- | --- | --- |
|  |  | **Low-dose**  **≥10^5^ to <10^6^ CFU** | **High-dose**  **≥10^6^ to <10^7^ CFU** |
|  | **(N=8)**  **n (%)** | **(N=8)**  **n (%)** | **(N=8)**  **n (%)** |
| Subjects with at least one adverse event | 8 (100.0) | 8 (100.0) | 8 (100.0) |
| Injection site pain | 3 ( 37·5) | 7 ( 87·5) | 5 ( 62·5) |
| Injection site erythema | 4 ( 50·0) | 3 ( 37·5) | 5 ( 62·5) |
| Blood fibrinogen decreased | 0 ( 0·0) | 1 ( 12·5) | 4 ( 50·0) |
| Headache | 3 ( 37·5) | 5 ( 62·5) | 2 ( 25·0) |
| Fatigue | 3 ( 37·5) | 2 ( 25·0) | 2 ( 25·0) |
| Injection site pruritus | 0 ( 0·0) | 0 ( 0·0) | 2 ( 25·0) |
| Injection site swelling | 1 ( 12·5) | 1 ( 12·5) | 2 ( 25·0) |
| Myalgia | 1 ( 12·5) | 4 ( 50·0) | 1 ( 12·5) |
| Lymphadenopathy | 2 ( 25·0) | 2 ( 25·0) | 1 ( 12·5) |
| Prothrombin time prolonged | 1 ( 12·5) | 2 ( 25·0) | 1 ( 12·5) |
| Upper respiratory tract infection | 0 ( 0·0) | 2 ( 25·0) | 0 ( 0·0) |
